# Current updates in tuberculosis

**DOI:** 10.1038/s41533-016-0007-5

**Published:** 2017-01-11

**Authors:** Gyanshankar Mishra

**Affiliations:** Department of Pulmonary Medicine, Government Medical College Nagpur, TB Center premises, Nagpur, Maharashtra 440003 India

The article by Giorgia Sulis and colleagues describes the recent updates in diagnosis and treatment of tuberculosis in a simplified way.^1^ However, some issues in the review require careful consideration.

The authors recognize XDR-TB (extensive drug-resistant tuberculosis) as MDR-TB (multidrug-resistant tuberculosis) cases with additional resistances to any fluoroquinolone and “any of the injectable drugs”, whereas the universally accepted definition of XDR-TB includes MDR-TB with resistance to any fluoroquinolone and “to at least one of the three second-line injectable drugs (capreomycin, kanamycin, and amikacin)”.^2,3^ Table 1 in the review also clearly mentions in the footnote that resistance to streptomycin alone does not qualify for the definition of XDR-TB.^1^ Thus, the definition of XDR-TB provided in the article needs to be reviewed as it has included “resistances to any of the injectable drugs” instead of “resistance to at least one of three second-line injectable drugs (capreomycin, kanamycin and amikacin).”

The article describes the treatment of M/XDR-TB primarily based on the recent World Health Organization (WHO) recommendations. It mentions that drugs like carbapenems and linezolid have been recently repurposed for use in M/XDR-TB and are “currently classified as group 5 drugs”.^1,4^ As per the recent WHO guidelines, linezolid has been elevated whereas carbapenems remain low, with the mandatory condition that they will only be used along with clavulanate. In this combination one has to be prepared to administer an injectable formulation through intravenous route for a prolonged period, which is a great limiting factor. The statement that these drugs are “currently classified as group 5 drugs” needs to be revised as they were previously classified as group 5 drugs.^2^ As per the new WHO classification, linezolid and carbapenem belong to groups C and D3, respectively. Also, this regrouping has removed the previously included clarithromycin.

The primary respiratory care physician has an important role in management of tuberculosis cases in the community. In developing countries like India, the standard of majority of the initial treatment provided for drug-sensitive tuberculosis still has a lot to improve.^5^ The current review will definitely be useful to the physician involved in primary respiratory care.
